# P383: Is infection control risk assessment required in hospital construction and renovation project?

**DOI:** 10.1186/2047-2994-2-S1-P383

**Published:** 2013-06-20

**Authors:** CHC Radley, TWM Josepha

**Affiliations:** 1 Infection Control Team, Queen Mary Hospital, Hospital Authority, HKSAR, Hong Kong

## Introduction

The nosocomial infection associated with the dispersal of microorganisms during construction has been reported in bone marrow transplant center, renal unit or intensive care unit.

## Objectives

To standardize the management of construction and renovation activities to reduce the infection risk as related to microorganisms from dust and debris.

## Methods

The infection control risk assessment (ICRA) for construction and renovation project, adopted from Australian hospitals, was modified and endorsed in July 2008. On aware of any building project, Hospital management would complete the assessment form to Infection Control Team. Class of barrier precautions would be determined by the Infection Control Team after multidisciplinary team assessment. Education on containment of dust was provided to contractors, whereas potential risks as associated with construction activities were delivered to staff. Completion of barrier precautions was checked before work commencement, whilst environmental cleanliness would be monitored continuously to ensure preventive measures adopted accordingly.

## Results

131 construction and renovation projects were followed by infection control team since July 2008. 67 (51%) construction areas were of high risk area whilst 75 (56%) projects required class 4 barrier precautions. Overall, no major environmental contamination was reported and air sampling results were within normal limits.

## Conclusion

Infection control team has an important role in standardization, collaboration, education and monitoring so that a formal approach to risk management becomes a part of construction and renovation activities within a health care facility.

## Disclosure of interest

None declared

